# The interpretation of low mood and worry by high users of secondary care with medically unexplained symptoms

**DOI:** 10.1186/1471-2296-12-107

**Published:** 2011-10-02

**Authors:** Christopher Burton, Kelly McGorm, David Weller, Michael Sharpe

**Affiliations:** 1 Centre for Population Health Sciences, University of Edinburgh, Teviot Place, Edinburgh, UK; 2 Scottish Primary Care Network, Royal Edinburgh Hospital, Edinburgh, UK; 3 Psychological Medicine Research, University of Edinburgh, Royal Edinburgh Hospital, Edinburgh, UK

## Abstract

**Background:**

Around 1% of adults are repeatedly referred from primary to secondary care with medically unexplained symptoms (MUS); many of these patients have depression and anxiety disorders which are unrecognized or inadequately treated. We aimed to investigate the ways patients with MUS and their General Practitioners (GPs) interpret low mood and worry, whether they regard them as depressive or anxiety disorders and how they relate them causally to symptoms.

**Methods:**

We carried out semi-structured interviews with 27 patients who had been repeatedly referred to specialists for MUS and their GPs and analysed transcripts by qualitative comparison. The analysis examined themes relating to low mood and worry, and their influence on symptoms. It drew on the concept of "otherness", whereby mental phenomena can be located either within the self or as separate entities.

**Results:**

Both patients and GPs acknowledged the presence of low mood and worry. They viewed low mood as either an individual's personal response to circumstances (including their physical symptoms) or as the illness called "depression"; only the latter was amenable to medical intervention. Worry was seen as a trait rather than as a symptom of an anxiety disorder. While low mood and worry were acknowledged to influence physical symptoms, they were considered insufficient to be the main cause by either the patients or their doctors.

**Conclusions:**

Patients with MUS who are high users of secondary care services interpret low mood and worry in ways which allow them to be discussed with professionals, but not as the cause of their physical symptoms.

## Background

A substantial proportion of primary and secondary care consultations are for physical symptoms which cannot be adequately explained by organic pathology. These are commonly termed Medically Unexplained Symptoms (MUS)[1] and are likely to arise from the interplay of physiological and psychological processes [2]. Around 1% of adults are repeatedly referred from primary to secondary care with symptoms which are found to be medically unexplained [3]. MUS are commonly accompanied by depression or anxiety [4,5]. Historically, MUS have been viewed as a manifestation of somatisation, the presentation of mental illness as physical symptoms [6]. This simple causal model underpins the therapeutic approach of reattribution which seeks to make the link between physical symptom and psychological cause [7] but is challenged by newer classifications [8].

In contrast to the simple causal model, implied by somatisation, patients with MUS make complex interpretations of the relationship between physical symptoms and emotional states, including low mood and worry [9,10]. More generally, people experiencing low mood and worry vary in the extent to which they consider these emotions pathological or normal [11,12]. In understanding the characterization of mental disorders such as depression, Karp [13], and subsequently Foster [14] developed the concept of "location" or "otherness", whereby mental phenomena are located either within the self or as separate "other" entities. In the case of depression, a patient locating low mood within the self might describe how "things get me down"; while another, viewing an apparently similar condition as 'other' might say "I suffer from depression". This notion of otherness is distinct from the view of depression as endogenous or reactive, in which it is the cause of depression that is viewed as occurring within the individual or in their environment rather than the phenomenon itself. These interpretations may be fluid rather than fixed [13] and can be seen as part of the drive to find meaning in experience [15,16].

We carried out a questionnaire and case-note review study of a large sample of patients who had been repeatedly referred to medical specialists with MUS. We found that around half of the patients met criteria for depression or anxiety but that many of them had received no treatment, or inadequate treatment, for these conditions [3]. As part of that larger study we conducted and analyzed semi-structured interviews of a subsample of patients and their general practitioners (GPs). Specifically we aimed to examine views about low mood and worry and their relationship to MUS in an attempt to understand why depression and anxiety are often neither recognized nor effectively treated.

## Methods

The researchers initially comprised a research nurse (KM), general practitioner (DW) and psychiatrist (MS). They were joined, for the data analysis, by another general practitioner (CB). This qualitative study was one part of a larger study of the role of MUS in referrals to specialists.

Patients were identified for the larger study from five general practices representing 30 GPs and 39,562 patients. The GPs were informed that the study would examine the role of MUS in repeated referral. We carried out linkage of a secondary care referral database to the practice databases followed by case note review carried out by the researcher (KM) in order to find patients who had been repeatedly referred to specialists. From this group we then identified all patients who had been referred to a specialist at least three times in the preceding five years because of physical symptoms, with at least two referrals resulting in a specialist concluding that the symptoms were not due to organic disease and thus fell within the broad category of MUS. These patients, and those in two control groups with different patterns of referral who are not included in this qualitative study were sent a questionnaire, which included the PHQ9 measure of depression, a measure of anxiety derived from the PRIME MD questionnaire and a brief measure of panic which are described in detail elsewhere [3]. Respondents whose most recent referral had been within the last few months, and was thus likely to be fresh in the memory, were invited by letter to take part in the interview study. Invitations were made irrespective of whether the most recent referral was for MUS or another 'medically explained' condition. The study was presented to patients as relating to referrals and the illnesses that lead to them, including broader aspects of health, rather than specifically either to MUS or depression and anxiety. The interviewees thus represented a convenience sample, drawn from a wider, well defined group of patients and we restricted analysis to those interviews for whom we obtained a paired GP interview. GP interviews were obtained by approaching the referring GP after the patient interview. While GPs were aware that MUS was of interest, the interview began by discussing the most recent referral; as not all of these were for MUS, GPs were encouraged to consider both the current problem and their broader knowledge of the patient.

Patient interviews were carried out by the research nurse (KM) in participants' own homes or by telephone and lasted between 30 and 120 (median 40 minutes). Several participants chose to have someone (typically a partner or relative), with them throughout the interview. Interviews were audio-recorded and subsequently transcribed for analysis. GP interviews were carried out by the research nurse either face-to-face or by telephone and were also recorded. Typically these were brief, median 8.5 minutes. GPs were not informed of the content of their patients' interviews.

The interviews followed a topic guide (Additional file 1). Patient interviews began with a discussion of the most recent referral and medical condition and then moved on to other experiences of illness and of being a patient. Issues relating to depression or anxiety were raised within the context of this discussion. There were also specific questions regarding current or past depression and anxiety and ways in which these might relate to physical symptoms. Interviews with patients and GPs were conducted without reference to patients' earlier responses to the questionnaire in order to obtain each participant's own account and maintain the researcher's independence from the diagnostic and treatment process. Towards the end of the analysis, data from the questionnaire (whether the patient met criteria for depression either at the time or in the past or been treated) was added to our reported findings. We did this to add context and not to challenge the validity of patients' statements; we chose not to do it initially, in order to concentrate on the patient's account without introducing the bias of a psychiatric diagnosis.

Analysis was conducted using a technique of qualitative comparison, however as the data were fully collected before rigorous analysis began it was not possible to feed emerging themes into later interviews, nor to prospectively seek disconfirming data. We used a phenomenological approach [17] to interpretation of the interviews, particularly those with patients, which centred on experiences and the meanings the participants made from them. For the analysis, the transcripts were read in depth by CB and KM in order to generate initial themes. These were then discussed and condensed to a final set of themes: the mapping from initial to final themes is shown in Additional file 2. We then reviewed the themes which related to depression and anxiety or to causality of symptoms in the light of relevant literature. Finally, CB re-read all transcripts in order to develop and refine the findings and to seek disconfirming data. These were then summarized, with examples, and discussed among all members of the research team through a series of one to one discussions rather than formal team meetings. Disagreements about interpretation of the data and choice of themes, were resolved through discussion by the multidisciplinary team.

The interview methods were tested in four pilot interviews which were not recorded.

All patients gave informed consent to the interview and analysis and the study had ethical approval from the Lothian Research Ethics Committee.

## Results

The five participating practices served areas which ranged from affluent to socioeconomically deprived. 45 patients were invited to take part in the interview study of whom 35 agreed. We carried out four pilot interviews without recording and data from these were not included in the analysis. 31 full interviews with patients were carried out and recorded and we obtained a matching GP interview for 27 of these. Our final sample was all of the paired patient and GP interviews.

The interviewed patients were aged between 34 and 64 years, 21 (78%) were female; 18 interviews were carried out in person and 9 by telephone. Based on their questionnaire responses, 9 (33%) participants met criteria for a current diagnosis of depression and a further 9 (33%) for an episode of depression in the last 5 years. Ten met criteria for a current anxiety disorder, either alone (4) or with depression (6). Twelve (44%) had recently received a prescription for an antidepressant and 16 (59%) had received at least one antidepressant prescription in the preceding five years. Patients most recent referrals were to a wide range of clinical specialties: six had been referred to general medicine, three each to orthopaedics, gynaecology, general surgery and urology, two to rheumatology, neurology and ophthalmology, and one each to rehabilitation, dermatology and ENT.

In keeping with the questionnaire responses, most patients described the experience of pervasive low mood or worry at some stage in their life; however they varied in describing these as 'feelings' or 'disorders' or both. In view of this we set a convention for reporting, by which patient's experiences were classified as low mood or worry rather than depression or anxiety, even where the questions may have included the latter terms. Patients appeared to vary in their location of these phenomena-either within the self or as "other", for example several of those describing low or unhappy feelings insisted that these did not represent depression, but that their low mood, for example shedding "tears of frustration", was a consequence of their physical limitation.

### Interpretation of low mood

Interviewees who reported low mood included both those who described this as "depression" and those who did not. Those who described low mood as depression, either currently or in the past, used terms indicating its otherness, for instance as "it", and as something they "suffered from". It was seen as something detached from current experience both in time and quality.

*I did have. For years and years and years I suffered from, I think it must have been, SAD [Seasonal Affective Disorder]. I used to cry, when there was nobody in, I used to cry from September until about March... cried my eyes out. And I didn't know why I was crying. And I went away to [medical herbalist], and er, I spoke to a lady there, and she gave me some pills and I was.., I don't know what they were, I took them and I've been fine since. That was years ago*.

Patient 19 (past depression prescribed antidepressant)

This otherness of depression did not necessarily imply a completely independent disorder. Depression could be still be seen as a process within a person's life, in terms of a reaction to illness and to the biographical disruption [16] that it brought.

*Yes. I've had to change my job. I used to be [a manager] and I was almost too busy. But now I'm not using my brain anymore. I'm more depressed and I have more time to think about it...... I have good days and bad. I can't believe it's all happening. But I just have to move on. The thought of non-improvement brings you down. Doctors suggest things, but there's no improvement*.

Patient 09 (depression on questionnaire prescribed antidepressant)

An important implication of describing depression in terms of otherness, either as an illness or a process, was that this made it worthy of medical recognition and intervention. Where depression had been recognized and treated (in most cases with antidepressant drugs), this was described in ways which suggested that it was appropriate and indicated a satisfactory doctor-patient relationship. However, some patients felt that doctors had failed to recognize their depression;

*No they haven't given me anything. I told them if they could bring me out of this depression, but they didn't give me anything. The GP and my thyroid doctor, I said to them and they didn't give me anything. My thyroid doctor said I was OK from my thyroid point of view. The GP gave me diazepam, but it didn't work so I didn't take them. I didn't feel better; I felt worse so I didn't take them. There must be something else*.

Patient 12 (depression on questionnaire, prescribed antidepressant)

Interviewees who experienced low mood but contested the label of depression included several who met our criteria for depression on the earlier questionnaire and who had been prescribed antidepressants either recently or in the past. Like those describing their low mood as depression, they interpreted their emotions in relation to events. However, by talking of low mood entirely as a reaction, rather than an "other" phenomenon, these patients normalized their experience and effectively removed it from the scope of medical intervention.

*All these years I haven't had depression. Sure I've been sad, and frustrated, but not clinically depressed; absolutely not. I was just reacting appropriately to the situation*.

Patient 04 (depression on questionnaire, no antidepressant)

GPs also talked of their patients' unhappiness and low mood as characteristics of the individual and their situation. Several described a line between low mood and clinical depression which might be crossed by the doctor diagnosing depression.

*And I think... but I don't think I've ever treated, or I don't think I've ever diagnosed clinical depression or given him anti-depressants. But I think his mood may have been a bit low at times when either his physical health has been not great, or he's been particularly hassled by neighbours and things, but no, I don't think I would call, no I've ever diagnosed him with clinical depression*.

GP 8, Patient 22 (depression on questionnaire, no antidepressant)

This hesitancy in labelling someone as having "clinical" depression is in keeping with the conservative approach to diagnosing depression found in other studies of GPs [18]. However, in some cases GPs were more explicit about labels of depression: "she is depressed and is prescribed antidepressants", GP 9 Patient 21 (depression on questionnaire, prescribed antidepressant).

### Interpretation of worry

In contrast to depression, anxious thoughts and worry were almost always located within the individual rather than as illness or disorders warranting medical intervention. Worry was presented as a reasonable response to current or future threat and justified by past events, particularly where there was a history of a delayed diagnosis or possible medical error in the past. Symptoms could be described as frightening, but the fear was justified by the severity, or possible significance, of the specific symptom.

*Yes, I worry about prostate cancer. I was involved in a study last year about prostate cancer, and a friend of mine had it. Every time I talk to him, I worry that I might get it. I also read in the newspaper that there is a test you can buy.... the PSA test. So I might get that done.... No, I don't have any symptoms, but it's in the back of my mind*.

Patient 03 (no questionnaire diagnosis or treatment)

Some interviewees recognized that they worried more about their health than most people, but even then this concern was seen as appropriate and within the normal range of experience.

*Some people need more reassurance than anything. Yes, reassurance that everything is OK. We all have the occasional lumps and bumps and it's nice to know that it's just fatty tissue or something and not a serious problem*.

Patient 10 (anxiety on questionnaire, prescribed antidepressant)

This was in contrast to the perceived behaviour of others whose anxiety and illness were seen as a matter of volition.

*But, I have no desire to be ill. I have a relative who's a hypochondriac, and I wonder how she can be so bothered being ill*.

Patient 15 (anxiety on questionnaire, no treatment)

In many interviews, worry was projected as a valuable attribute, for instance ensuring safety for the individual or her family. Even when worry was seen as present it could still be accompanied by statements which attempted to project the patient's integrity and credibility.

*Oh no no no, that's the important thing. I never, ever for a minute am saying, you know, that 'I've got throat cancer'. But I'm the sort of person who says, 'I could have throat cancer' because people get throat cancer, and one of those people could be me. And please God it's not me, and I'm not for a minute going to assume that it's going to be me.. It's just another fact of life. No no, I'm not. Honestly I'm not. I'm not that type*.

Patient 25 (no questionnaire diagnosis or treatment)

Only one interviewee described anxiety both in the external context of mental illness and the internal context of a personal characteristics.

*I had a nervous breakdown about 15 years ago. It all came to a head with the death of a parent, and I had a relationship problem, and work problems and I hadn't had a holiday in years. If I had known then what I know now, I would have done more. Now there seems to be a greater level of awareness. I don't think I'm an anxious person, now, but I do like to keep on top of things and don't like to muck about*.

Patient 15 (anxiety on questionnaire, no treatment)

GPs rarely described their patients as being diagnosed with anxiety disorders and did not use psychiatric diagnoses such as Generalized Anxiety Disorder or Panic Disorder explicitly. However they did appear to recognise the presence of anxiety in their patients but struggled to describe it in more than uncertain terms:

"chronic anxiety. I think he has obsessive trait and sort of, chronic anxiety which is sort of quite a powerful mixture"

GP18, Patient 25 (no questionnaire diagnosis or treatment)

When GPs described anxiety more clearly, they tended to do so either as a personal characteristic-drawing on their knowledge of the patient and their family over time-or as a process rather than a specific disorder.

*Her Mum's got similar problems to [patient] actually, sort of bronchitis, anxiety and agoraphobia. But [patient]'s not as bad as that, but she is an anxious lady*.

GP 20, Patient 17 (past depression prescribed antidepressant)

*If she is anxious she focuses more on her health and when she worries about her health, it makes her more anxious, so she gets herself into quite a vicious circle*.

GP13 Patient 07(anxiety on questionnaire, past antidepressant)

### Causality in relation to low mood and worry

The notion of causality was common to most accounts: for instance, pain or isolation could cause low mood, or the specific threat of a physical symptom could cause worry. Patients' accounts of causality were similar between depression and anxiety

*I really don't know because I'm actually no' bad at managing the pain myself, the now em... when I say I am good at managing it, after a while it does get you down, ken. You did sit and greet [weep] about it, I do have a cry about it now and again because I get so frustrated*.

Patient 28 (past depression, past antidepressant)

*Well I mean I get anxious about my health but I get anxious about my health because of my health, you know it's not some convoluted other thing in my psyche. Ah no it's.. not at all*.

Patient 25 (no questionnaire diagnosis or treatment)

While the interviews all contained explanations in which physical symptoms led to worry or low mood, and many described low mood in relation to difficulties in life, very few contained specific attributions in which emotions led directly to physical symptoms and even then it was only something discovered with hindsight:

*So... ah... it's hard. And my daughter had miscarried and so it was 10 years of stress, and I think that's what they dealt with in the beginning. So it could've been a condition I've got into, triggered by stress, which I don't know is happening. Because when the phone used to ring I used to go 'Uhhh what's happened now?' But whether it's still a response that lives with me, I don't know it's happening, maybe it is, like a nervous stomach or something like that-it could well be*.

Patient 27 (no questionnaire diagnosis or treatment)

Some GPs described bidirectional models of causality in which physical ill health and psychological distress could each lead to the other; however, these were described as personal reflections rather than as explanations which they used with patients.

*I was thinking about this, because she is a lady I really know quite well, and she's really had a lot of ill health in the past, she's had breast cancer and things, so she's had a lot to cope with. And I think it's difficult to know whether her physical health problems get her down, or... it's the old story... or she's down and her physical problems seem worse. And I suspect it's a bit of both*.

GP 21, Patient 19 (past depression on antidepressant)

Despite this, others used much more rigid statements, often to deny a psychological component, for instance "You don't imagine kidney stones". On the few occasions when GPs used the term "psychosomatic" it was in a way which suggested it was an unacceptable label term for their patient, and none used the term medically unexplained symptoms-indeed one corrected himself when about to

*She does tend to present with unexpl.. with difficult to explain symptoms, with back pain and joint pains which then you can't really find any objective cause for. And um, she certainly talks a lot about her arthritis, which she doesn't actually have any arthritis on x-ray. So I think that's her way of formulating a pain, which I'm sure is there. So I'm quite happy to go along with that*.

GP 20, Patient 17 (no questionnaire diagnosis or treatment)

## Discussion

### Summary of main findings

The patients who had been repeatedly referred from primary to secondary care with MUS and their GPs both acknowledged that the patients had low mood and worry. They viewed the low mood as either an individual's personal reaction to circumstances (including their physical symptoms) or as an illness called "depression"; only the latter was seen as amenable to medical intervention. Worry was seen as a trait rather than a symptom of an anxiety disorder. While low mood and worry were acknowledged to influence physical symptoms, they were considered insufficient to be the main cause of these by either patients or their GPs.

### Strengths and limitations of this study

This study involved patients who had been referred from primary to secondary care with medically unexplained symptoms on at least two occasions who were initially identified using the computer databases of the healthcare system. While this reduced the risk of recruitment bias, as might happen if GPs invited only patients they felt comfortable with, it also reduced the opportunities for purposive sampling of specific cases. By limiting ourselves to these higher healthcare users with MUS we focused on a group of patients likely to have specific characteristics and behaviours which may not be representative of all patients with MUS. While the sampling method was not purposive, our sample included referrals to a wide variety of clinical specialties, demographic and socioeconomic diversity and a mixture of patients with and without current or past mental health problems and treatments. Inspection of the later interviews suggests that saturation was being reached in the key themes of this paper.

While the questionnaire results were not used in selecting interviewees and were not made available for the initial interpretation, this information was added later to add context to the findings. We did not carry out formal psychiatric interviews-however the questionnaire items used have good sensitivity and specificity in this kind of population [19]. Although the analysis was conducted after the interviews were all completed, there was discussion of the emerging findings and the interview content between the interviewer (KM) and two of the authors (DW and MS) as data was being collected. Nevertheless this meant that we were unable to test emerging hypotheses explicitly and weakens the strength of our analysis. The GP interviews were typically short and the issues of location, causality or attribution of symptoms were not directly raised. Despite this and the fact that some comprised very brief answers only, several contained evidence of uncertainty on the GPs part suggesting that the interviewee was engaged in reflective thought rather than just reciting the answers that might be expected.

By using the concept of location of mental phenomena [13] or the "otherness" of mental disorders [14] in the analysis we were able to build our analysis on existing research and theory. Nevertheless, the inability to test hypotheses about location and causality in later interviews represents a substantial weakness and should be tested in further studies.

### Relationship to previous studies

We are not aware of prior qualitative studies specifically examining the labelling of depression and anxiety in the context of patients with MUS. In general, GPs' diagnosis of depression is negotiated over time [20] rather than applied immediately. However, our patients had a history of MUS lasting several years, so it is unlikely that diagnoses of depression were still evolving. Other factors may have acted against making a formal diagnosis. Patients with depression endeavour to "maintain face" [21] and comparable "moral work" [22] is carried out by GPs who present themselves as supporting and understanding patients even when that is difficult [23]. Patients with medically unexplained symptoms-in which their experience of abnormality is at odds with professionals' findings of normal tests-face particular difficulties in maintaining their integrity as credible individuals [24]. Our findings of patients' and GPs' willingness to understand low mood as something explicable for a person in adversity, and to reframe anxiety as a potentially protective personal characteristic, are in keeping with a joint attempt to accommodate these otherwise problematic emotions in a way which avoids showing weakness or threatening the doctor-patient relationship.

Patients with MUS have complex frameworks of causality [25] with multiple components including psychosocial factors [9]. They are, however, typically wary of discussing emotional issues unless they are confident that their doctor will continue to deal with their physical problems [10]. The reluctance by GPs to label symptoms as medically unexplained has been noted elsewhere [26] and in the absence of readily available treatment or explanation, GPs appear to prioritize the maintenance of the doctor-patient relationship, either by offering personal support or by repeating the medical "rituals" of examination and referral [27]. We found little evidence of GPs adhering to a psychiatric model of somatisation, whereby physical symptoms are seen as explicit representations of emotional distress. This study was not designed to critique that model per se, but it does suggest that while GPs are aware of emotional distress in their patients they lack plausible frameworks to link this to physical symptoms in a way which is acceptable to patients. Recent work suggests that patients own explanations of their mental health should be the starting point for this discussion [28].

### Implications for clinical practice and research

This study adds plausible mechanisms to our quantitative findings that depression and anxiety commonly go unrecognized and untreated in patients repeatedly referred to hospital for MUS [3]. We found little evidence of patients not recognizing their emotional distress, nor of GPs being unaware of it. Instead, our findings suggest that keeping low mood or worry as personal responses to adversity, or as traits, avoids the difficulties of attributing physical symptoms to a mental disorder, for both patient and doctor.

Given our findings, combined with the lack of benefit from reattribution and the low adherence to antidepressant treatment in patients wary of taking it [29], we believe that dealing with depression or anxiety by insisting on a psychiatric diagnosis and applying a psychosomatic model for the cause of physical symptoms is unlikely to be productive. Rather, two alternative approaches are needed: first doctors should recognize and deal with depression and anxiety, not as the cause of MUS, but as important and treatable co-factors in the distress which results from them. Second doctors should develop explanations for MUS which are compatible with patients' self perceptions [30]. Effective cognitive behavioural therapy models have been developed which take this dual approach [31], but briefer interventions are urgently needed which can be deployed in primary or intermediate care. We are currently developing an intervention which explains and addresses symptoms in terms of cyclical processes (both physiological and psychological) rather than direct linear cause.

## Conclusions

Patients with MUS interpret low mood and worry in ways which permit them to be discussed with their doctor but which negate psychosomatic causality. Unless physicians use explanatory models for MUS which permit depression and anxiety as a co-factor in physical symptoms rather than their cause, we expect that under-recognition and inadequate treatment of these disorders will continue.

## Competing interests

The authors declare that they have no competing interests.

## Authors' contributions

MS conceived and designed the study, KM conducted the interviews with supervision from MS and DW, CB and KM carried out the analysis CB drafted the paper and all authors contributed to the analysis and interpretation. All authors read and approved the final manuscript.

## Pre-publication history

The pre-publication history for this paper can be accessed here:

http://www.biomedcentral.com/1471-2296/12/107/prepub

## Supplementary Material

Additional file 1 **Interview topic guide**. Click here for file

Additional file 2 **Mapping of initial to final themes**. Click here for file
