# Supplementary material for: The interpretation of low mood and worry by high users of secondary care with medically unexplained symptoms
Source: BMC Fam Pract. 2011 Oct 2;12:107. doi: 10.1186/1471-2296-12-107 (PMC3197491; doi:10.1186/1471-2296-12-107)
Supplement: Additional file 1 — Interview topic guide. [file 1471-2296-12-107-S1.DOC]

**Additional file 1: Interview topic guide**

**1. Patient Interview**

**Section A: discussion of symptoms leading to referral and of patient expectations:**

*Introduction “I want to start off by asking you about your recent health concerns.”*

- Can you tell me about your recent symptoms or health concerns?
- Were there other things going on in your life that may have been affecting your health?
- What led you to see your GP about it?
- What did you hope would happen when you went to see your GP?
- What did you expect would happen when you went to see your GP?
- What did the GP say about referring you to hospital on this occasion?

**Section B: experience of referral / waiting**

*Introduction* *Now I’d like to go on to talk about what might follow on from this consultation with the GP…*

- Have you been given an appointment to see the hospital doctor? (Or if not, have you been told how long you may have to wait to be seen?); how do you feel about this waiting period?
- What do you hope to happen when you see the hospital doctor?
- What do you expect to happen when you see the hospital doctor; do you think being referred to hospital will resolve your problem?

**Section C: emotional well being**

*Introduction: I’d like to talk about your emotional well being at this time as both physical and emotional factors play an important role in your health;*

- do you think you have any:
  - Particular fears or worries about your health which may be impacting on your health?
  - Anxiety affecting your health?
  - Depression affecting your health?
- Are you currently having any treatment for anxiety, depression or stress?
- Do you feel that you would benefit from such treatment?

**Section D: use of health care services**

*Introduction: I’d like to talk to you about your use of health care services*

- How often do you usually see your GP / attend hospital services or clinics
- Have you had any problems with the care you have received from the health service?
- Other than being referred to hospital, are there any other types of care or support (not necessarily medical) that you think may help you at this time?

**Section E: any other areas, conclusion**

**2. GP Interview**

**The topic guide for GP interviews follows broadly the same sections as patient interview. The relevant section on emotional well being is as follows:**

- Do you think the following may be affecting this patient's health:
  - Anxiety?
  - Depression?
  - Fears about their health?
- Is this patient on treatment for anxiety and/or depression? If not, do you think they might benefit from such treatment?
- Other than being referred to hospital, are there any other types of care or support (not necessarily medical) that you think may help this patient at this time?
