# Supplementary material for: The interpretation of low mood and worry by high users of secondary care with medically unexplained symptoms
Source: BMC Fam Pract. 2011 Oct 2;12:107. doi: 10.1186/1471-2296-12-107 (PMC3197491; doi:10.1186/1471-2296-12-107)
Supplement: Additional file 2 — Mapping of initial to final themes. [file 1471-2296-12-107-S2.DOC]

**Additional File 2 : Mapping of initial to final themes.**

| **Initial Theme** | **Patient[[1]](#footnote-2)** | **Doctor** | **Final theme** | **External evidence** |
| --- | --- | --- | --- | --- |
| **Depression / low mood** |  |  |  |  |
| Person with depression | ✓ | ✓ | Interpretation of low mood | Location – external to self (disorder) or within self (characteristic) |
| Becoming low in mood | ✓ |  |
| Person who is sad |  |  |
| **Anxiety / worry** |  |  |  |
| Person with anxiety |  | ✓ | Interpretation of worry |
| Becoming worried | ✓ |  |
| Person who is a worrier | ✓ | ✓ |
| **Causality** |  |  |  |  |
| Stress and illness | ✓ | ✓ | Causality between low mood / worry and physical symptoms | Literature on attribution |
| Symptoms cause anxiety / depression | ✓ | ✓ |
| Anxiety / depression cause symptoms | ✓ | ✓ |
| **Other** |  |  |  |  |
| Showing / hiding anxiety and depression | ✓ |  | Not included in this analysis. | Work of managing MUS[[2]](#footnote-3) |
| Challenging patients |  | ✓ |
| Knowing the patient |  | ✓ |

1. Columns indicate whether this theme appeared in patient or doctor interviews [↑](#footnote-ref-2)
2. This theme is not reported in detail, however work reporting similar themes is included in the discussion. [↑](#footnote-ref-3)
